# Clinical performance of biodegradable versus permanent polymer drug-eluting stents: A meta-analysis of randomized clinical trials at long-term follow-up

**DOI:** 10.3892/etm.2015.2293

**Published:** 2015-02-13

**Authors:** QI WANG, YU ZHOU, TONG QIAO, MIN ZHOU

**Affiliations:** Department of Vascular Surgery, Drum Tower Clinical Medical College of Nanjing Medical University, The Affiliated Drum Tower Hospital of Nanjing University Medical School, Nanjing, Jiangsu 210008, P.R. China

**Keywords:** percutaneous transluminal angioplasty, endovascular therapy, drug-eluting stent, biodegradable polymer, permanent polymer, outcome analysis, meta-analysis

## Abstract

Several types of biodegradable polymer drug-eluting stents (BPDES) have been used for percutaneous transluminal angioplasty; however, the safety and efficiency of these BPDES have not been fully evaluated. A meta-analysis was, therefore, conducted to compare the clinical performance of BPDES with that of permanent polymer drug-eluting stents (PPDES) in unselected patients with coronary stenosis. PubMed, Web of Science, Medline and The Cochrane Library were searched for randomized clinical trials (RCTs) from January 2005 to January 2014. Trials that compared BPDES with PPDES in patients with coronary stenosis were considered. Twelve RCTs with a total of 15,938 patients with coronary stenosis were included in this meta-analysis. No significant difference was found between the two arms in the incidence of major adverse cardiac events (MACE) and definite or probable stent thrombosis (DpST) at the one-year follow-up (P>0.10). The use of BPDES, however, showed a tendency towards a lower risk of MACE (P=0.09) and a beneficial effect by reducing DpST episodes (P=0.04) at long-term follow-up, particularly when compared with the incidence of DpST at the one-year follow-up. BPDES also tended to be associated with a decreased late lumen loss in patients with coronary stenosis [instrumental variable =−0.04; 95% confidence interval =−0.08–0.00; P=0.05). In conclusion, the one-year outcomes following drug-eluting stent implantation showed BPDES were noninferior to PPDES in unselected patients with coronary stenosis. Long-term clinical outcomes, however, indicated that BPDES appeared to a present a lower risk of MACE and DpST.

## Introduction

Over the past few decades a large number of patients have undergone percutaneous transluminal angioplasty (PTA), but they have also suffered a high risk of restenosis following PTA (30–50%) (1), which has been a serious problem in interventional cardiology. The emergence of bare-metal stents and subsequent drug-eluting stents (DES) eased this problem, with the latter, in particular, greatly reducing the restenosis risk to ~10% (2). With an increasing number of patients receiving DES and more data available from long-term follow-up studies, the safety of these devices has been associated with a rise in the rate of late stent thrombosis (LST) and very late stent thrombosis (VLST) (3,4). Numerous animal and human studies have demonstrated that the hypersensitive reaction to durable polymers on DES may play a major role in the DES-induced inflammation and delayed vascular healing, which subsequently causes LST and VLST following intervention (5,6). Given these problems, biodegradable polymer drug-eluting stents (BPDES) emerged, which are equipped with biodegradable polymer drug carriers that degrade at the same time as the drug is released until they completely disappear and only the stent remains. This type of DES can, therefore, reduce or eliminate the stimulatory effect of the polymer on the vessel so as to, theoretically, reduce the incidence of LST and VLST (7); however, the degradation of biodegradable polymers may succumb to negative factors, some of which influence the velocity of degradation, either by accelerating it or slowing it down (5). To further study the influence of biodegradable polymers on stent performance, a large number of controlled clinical studies have been conducted to observe the clinical efficacy of BPDES. According to a meta-analysis of 10 trials, BPDES significantly reduced late lumen loss (LLL) and target vessel revascularization (TVR) but without clear benefits on mortality, myocardial infarction (MI) and LST rates when compared with permanent polymer drug eluting stents (PPDES) at the one-year follow-up (8). Another meta-analysis of 22 clinical trials did not show that BPDES were better than PPDES regarding the incidence of definite or probable stent thrombosis (DpST) at one year following implantation (9); however, there has been no meta-analysis comparing the clinical outcomes of BPDES and PPDES at >1 year follow-up. In addition, the performances of various BPDES with different eluting drugs have not been fully evaluated; therefore, the present meta-analysis was conducted to try to rectify these omissions.

## Materials and methods

### Eligibility criteria and search strategies

To be included in this meta-analysis, trials were required to meet the following criteria: i) Randomized clinical trials (RCTs) comparing a DES with a biodegradable polymer and a DES with a permanent polymer in patients undergoing percutaneous coronary intervention; ii) enrolment of >50 patients with available follow-up data for at least one of the clinical end-points or angiographic end-points at mid-term (≤9 months) and/or long-term (≤16 months). PubMed, Web of Science, Medline and The Cochrane Library were searched between January 2005 and January 2014 for RCTs on BPDES. The PubMed search strategy was formulated as follows: (‘biodegradable polymer’ OR ‘bioabsorbable’) AND (‘permanent’ OR ‘durable’) AND ‘clinical trials’. This search strategy was translated to the corresponding vocabulary of Medline, Web of Science and Cochrane Central Register of Controlled Trials. No language restriction was applied and the search was kept updated until January 2014.

### Study selection and risk of bias

To select trials, the following steps were performed following trial identification by the main search: i) Exclusion of duplicates; ii) screening and selection of abstracts; iii) assessment for eligibility through full-text articles; iv) final inclusion. One author followed steps i) to ii) and another two authors followed steps iii) to iv) independently. Disagreements were resolved by discussion.

Three authors independently assessed the risk of bias with the components recommended by the Cochrane Collaboration: Random sequence generation; allocation concealment; blinding of participants and personnel; blinding of outcome assessment; incomplete outcome data; selective reporting and other sources of bias (10). Trials with a high or unclear risk for bias for any one of the first two or the fourth components were identified as trials with a high risk of bias; otherwise, they were identified as trials with a low risk of bias. Trials were excluded if they lacked a clear statistical analysis or did not adjust for potential confounders.

### Data extraction

Two authors independently performed data extraction on the trials. Any differences found were resolved by discussion. The information was collected regarding the main clinical characteristics (first author, year of publication, trial acronym, event location, type of stent, number of participants and lesions, age and gender, proportion of patients under the risk factors of smoking, hypertension and diabetes, proportion with previous MI, duration of treatment with thienopyridines and the maximum follow-up period) and angiographic characteristics (location of target lesion, reference vascular diameter, minimal lumen diameter and target lesion length). For both biodegradable polymer stent groups and permanent polymer stent groups, major adverse cardiovascular events (MACE), including cardiac mortality, MI or TVR were defined as the primary clinical outcomes. Other clinical or angiographic outcomes of interest included DpST, LLL in the stent and stenosis of lumen diameter (SLD) in the stent. In order to better compare the differences between short- and long-term follow-up results of BPDES and PPDES, the results are firstly classified into the one-year follow-up group (with a period of 12 months) and the long-term follow-up group (with a period of >12 months) according to the length of the follow-up period.

### Statistical analysis

Statistical analysis was performed using Review Manager (version 5.2; Cochrane Collaboration, Copenhagen, Denmark). Summary statistics of dichotomous variables were presented as odds ratios (OR) and 95% confidence intervals (95% CI). Continuous variables were calculated as weighted mean difference with 95% CI. Following data pooling, statistical heterogeneity across trials was identified and evaluated by Cochrane Q χ^2^ and I^2^ statistics. Trivial heterogeneity was considered for P-values >0.1 or I^2^<50%, and a fixed-effect model would be used. A random-effect model replaced the fixed-effect model if P<0.1 or I^2^>50%, which suggested substantial and significant heterogeneity. The likelihood of publication bias was assessed graphically by generating a funnel plot for the primary end-points and angiographic outcomes. Subgroup analysis was performed based on the eluting drugs. Following the completion of data analysis, the GRADEPro system (Cochrane Informatics and Knowledge Management, London, UK) was used for the scoring of the main analysis results to assess the value of each.

## Results

### Trials and trial characteristics

**Figure 1:**
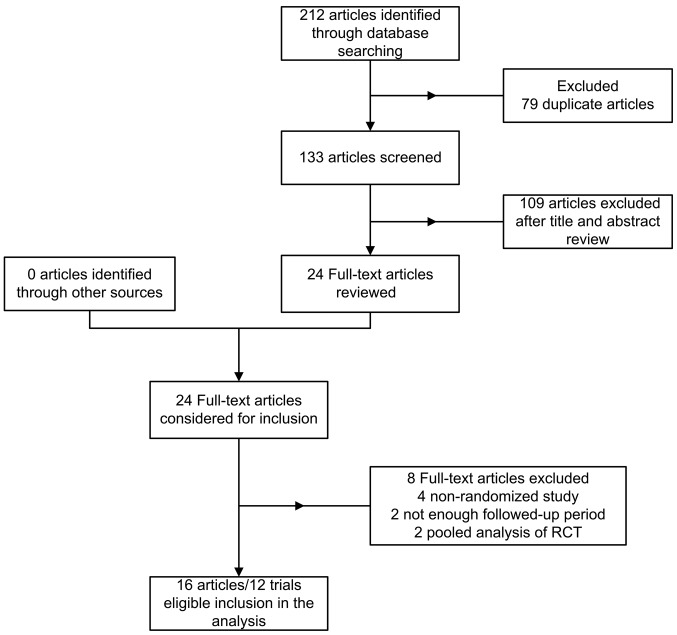
Flow chart of trial selection.

**Figure 2:**
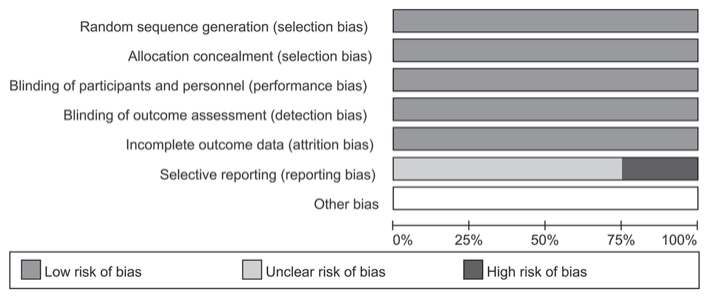
Risk of bias graph. Judgement of the review authors about each risk of bias item for each included trial. Each risk of bias item is presented as a percentage across all included studies.

Seventy-two papers were identified from PubMed, 69 from Web of Science, 17 from The Cochrane Library and 54 from Medline. A total of 79 duplicates were excluded leaving 133 studies identified by the main search. A further 109 papers were excluded by reading the titles and abstracts so that 24 potentially relevant papers were identified. Finally, a total of 16 articles concerning 12 RCTs (11–26) with a total of 15,938 patients with coronary stenosis were included in the current meta-analysis. A flow chart showing trial selection is shown in Fig. 1. Among these patients, BPDES were used in 8,643 patients while PPDES were used in 7,295 patients. The main demographic and clinical characteristics of the included trials are summarized in Tables I and II. No significant difference was identified in the main characteristics of patients between the biodegradable polymer (BP) and permanent polymer (PP) groups. The mean age of the participants in individual trials ranged from 58 to 69 years with males representing the majority. The percentage of diabetic patients was 29.1% among the BP group and 29.6% among the PP group. The minimum duration of thienopyridine therapy following stent implantation was variable between these trials; three months in two trials (18,22), six months in five trials (11,14,15,19–21) and 12 months in five trials (16,17,24–26). The maximum follow-up period was from nine to 60 months. Data extracted from trials with a >16 month follow-up period were defined as long-term outcomes and were analyzed as an individual group. The risk of bias for all included studies is shown in Fig. 2. The random sequence generation and allocation concealment of RCTs were well described. It was found that none of the trials blinded participants and personnel. As blinding of participants and personnel had little influence on outcome assessment, it was considered an insignificant and low-risk source of bias. Three trials (19,24,26) were judged to have a high risk of reporting bias due to the fact that one of the outcomes of interest in the study was reported incompletely; thus, it could not be included in this meta-analysis. Other trials were considered to have an unclear risk of bias on reporting bias as no clear information was found to judge them either low risk or high risk.

### Clinical outcomes at one-year-follow-up

#### MACE

**Figure 3:**
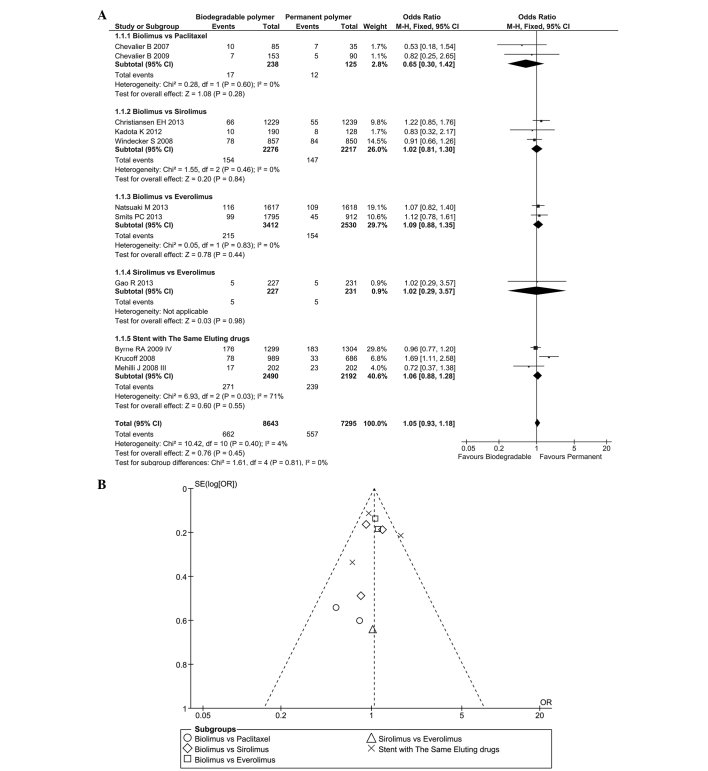
(A) Forest plot and (B) funnel plot of the study of the incidence of major adverse cardiac events in biodegradable polymer vs. permanent polymer stents at the one-year follow-up. CI, confidence interval; M-H, Mantel Haenszel; SE, standard error; OR, odds ratio; df, degrees of freedom.

MACE data were acquired from 11 RCTs between nine and 16 months following the stent installation. As there was no significant heterogeneity (P=0.40, I^2^=4%), a fixed effect model was used (total OR=1.05, 95% CI=0.93–1.18, P=0.45; Fig. 3A) and the results revealed that the incidence rate of MACE was similar in the BP and PP groups at the one-year follow-up. This outcome was observed in each subgroup, and the difference between subgroups was low (I^2^=0%). A funnel plot was used to assess the likelihood of publication bias, as presented in Fig. 3B.

#### DpST

**Figure 4:**
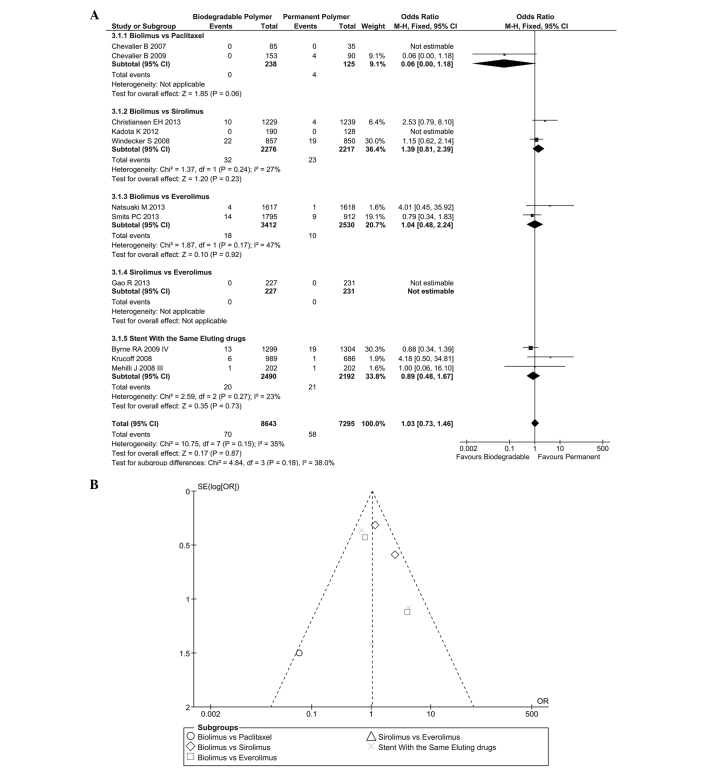
(A) Forest plot and (B) funnel plot of the study of the incidence of definite or probable stent thrombosis in biodegradable polymer vs. permanent polymer stents at the one-year follow-up. CI, confidence interval; M-H, Mantel Haenszel; SE, standard error; OR, odds ratio; df, degrees of freedom.

DpST at the one-year time-point is reported in Fig. 4A. No significant heterogeneity was found (I^2^=35%; P=0.15) among these trials so a fixed effect model was selected. No significant difference was found between these two groups for DpST (OR=1.03, 95%CI=0.73–1.46, P=0.87). The subgroup analysis of different eluting drugs showed comparable results and inter-group heterogeneity was low (I^2^=38%). The likelihood of publication bias is shown in Fig. 4B.

### Clinical outcomes at long-term-follow-up

#### MACE

**Figure 5:**
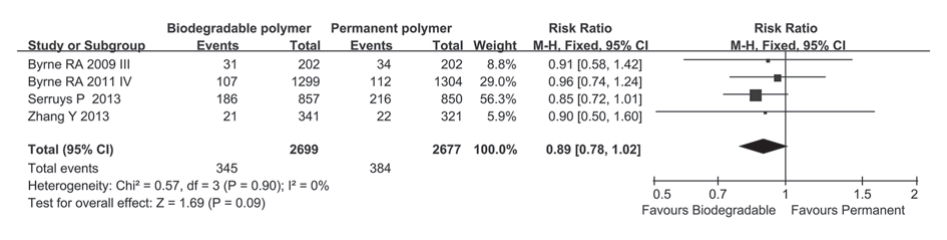
Forest plot of the study of the incidence of major adverse cardiac events in biodegradable polymer vs permanent polymer stents at long-term follow-up. CI, confidence interval; M-H, Mantel Haenszel; df, degrees of freedom.

MACE data were acquired from four RCTs regarding long-term follow-up (Fig. 5). No heterogeneity was found (P=0.90, I^2^=0%) among these trials so a fixed effect model was selected (total OR=0.89, 95% CI=0.78–1.02, P=0.09). Results showed no significant difference between these two groups.

#### DpST

**Figure 6:**
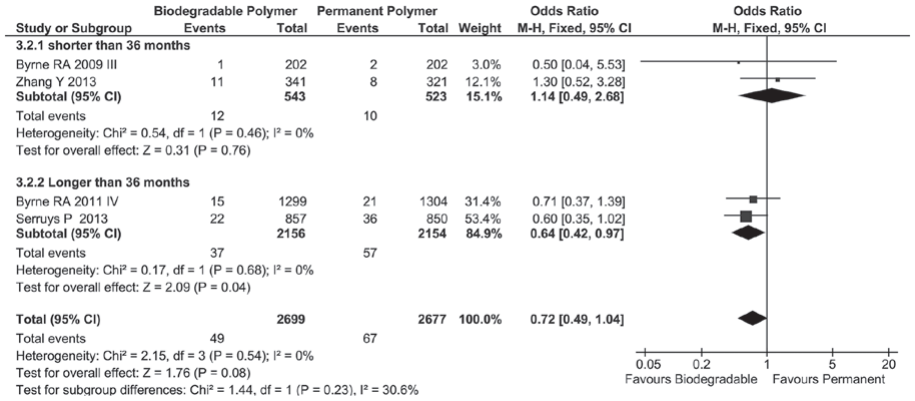
Forest plot of the study of the incidence of definite or probable stent thrombosis in biodegradable polymer vs. permanent polymer stents at long-term follow-up. CI, confidence interval; M-H, Mantel Haenszel; df, degrees of freedom.

Regarding long-term follow-up, the incidence of DpST in these two groups was similar but the BP group showed a tendency to reduced DpST compared with that in the PP group (OR=0.72, 95% CI=0.49–1.04, P=0.08; Fig. 6) and no heterogeneity was found among these four trials (I^2^=0%). Subgroup analysis showed a statistically significant difference between the BP and PP groups at >36 months follow-up (OR=0.64, 95% CI=0.42–0.97, P=0.04).

### Angiographic outcomes

#### LLL in stent

**Figure 7:**
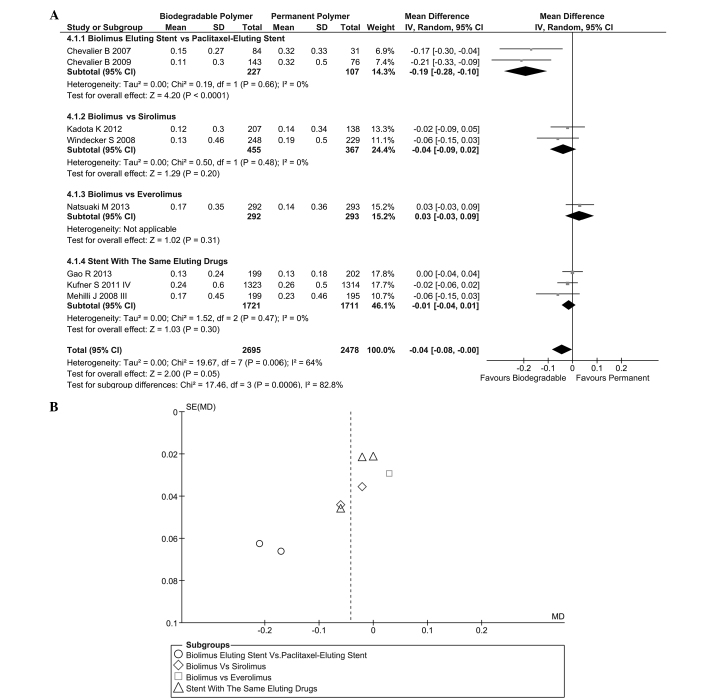
Forest plot (A) and funnel plot (B) of the data about the late lumen loss in biodegradable polymer vs. permanent polymer stents at one-year follow-up. CI, confidence interval.

Regarding the 6–12-month follow-up, results of LLL in stent were acquired in eight trials, as shown in Fig. 7A. Heterogeneity was found (I^2^=64%, P=0.006) among these trials and a randomized effect model was selected. The results [instrumental variable (IV)=−0.04, 95% CI=−0.08–0.00, P=0.05] indicated that the difference between the BP and PP groups was considered to be statistically significant. Subgroup analysis showed that the biolimus-eluting stent (BES) was superior to the paclitaxel-eluting stent (PES) (IV=−0.19, 95% CI=−0.28 to −0.10, P<0.001). A funnel plot of this result is shown in Fig. 7B.

#### Stenosis of lumen diameter (SLD)

**Figure 8:**
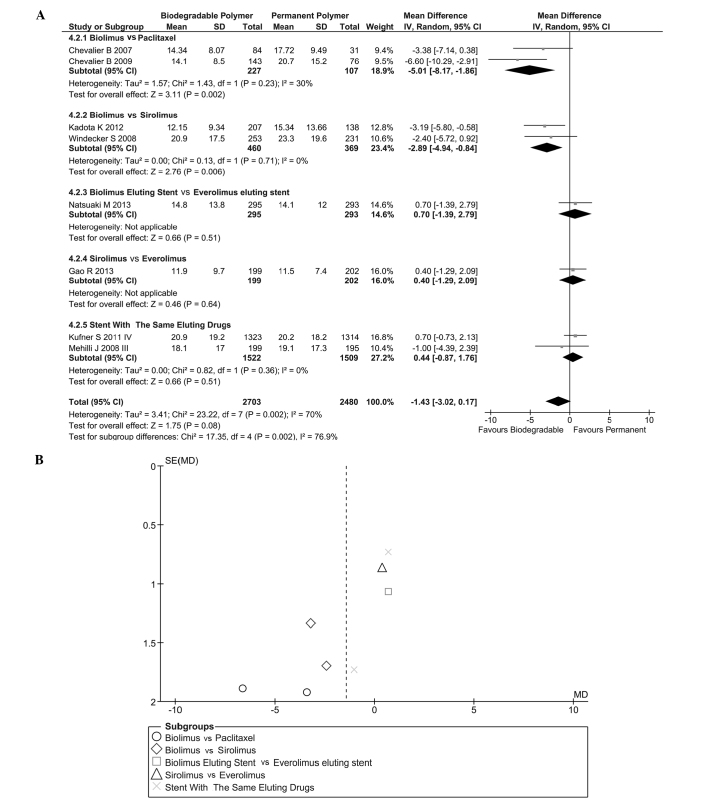
Forest plot (A) and funnel plot (B) of the data about the stenosis of lumen diameter in biodegradable polymer versus permanent polymer stents at one-year follow-up. CI, confidence interval; SD, standard deviation; df, degrees of freedom.

SLD (in stent) is shown in Fig. 8A. Heterogeneity was found (I^2^=70%, P=0.002) and a randomized effect model was used (IV=−1.43, 95% CI=−30.2–0.17, P=0.08). Subgroup analysis indicated that BES could effectively decrease the severity of SLD in stent compared with PES (IV=−5.01, 95% CI=−8.17 to −1.86, P<0.01) and sirolimus-eluting stent (SES) (IV=−2.89, 95% CI=−4.94 to −0.84, P<0.01). This result indicated that a BES was more effective in reducing SLD in stent than other drug-eluting stents. Heterogeneity between these groups was significant (I^2^=94.6%). The likelihood of publication bias was assessed by funnel plot (Fig. 8B).

#### Evaluation of results

Following analysis of the data from RCTs by Review Manager, the GRADEPro system was used to evaluate the results. In conclusion, the quality of each result concerning long-term follow up was high. Details of the evaluation are shown in Table III.

## Discussion

DES are a major breakthrough in the field of PTA, since they have markedly reduced the rate of acute stent thrombosis and the requirement for repeated revascularization procedures compared with bare-metal stents (27,28). As the use of DES for artery stenosis has increased, an increasing amount of attention has been paid to the potential inflammatory response, which occurs due to the polymers used for the delivery of the anti-restenotic agents (7). BPDES were designed to solve this problem. There have been clinical trials evaluating the safety and efficacy of BPDES but the present study, to the best of our knowledge, is the first meta-analysis to compare the clinical performances of BPDES and PPDES in patients with coronary stenosis at short- and long-term follow-up periods.

In this analysis, the RCTs that were considered included numerous countries and regions; therefore, they covered the different races of the world. The analyzed BPDES group contained three types of stents with different eluting drugs: Sirolimus (11,17,21,26), biolimus (14–16,18,22,24,25) and paclitaxel (19). The PPDES group was also equipped with three different drugs: Everolimus (17,22,24), paclitaxel (14,15,19) and sirolimus (11,16,18,21,25,26).

The analysis of clinical events at the one-year follow-up showed that the incidence of clinical events due to BPDES was not significantly different from that due to PPDES. More accurately, the BPDES were noninferior to PPDES in safety profile one year following DES implantation. In the present study, the incidence of various clinical events in the analyzed RCTs was maintained at a low level, which provided evidence for the safety of modern stents.

Nine RCTs reported angiographic outcomes. It was found that the degree of LLL in patients receiving BPDES was significantly lower than that in patients receiving PPDES. The Nobori I (14) and Nobori I phase 2 trials (16) reported an advantage in using BPDES, while other trials did not identify any significant difference between BPDES and PPDES. In addition to LLL, the analysis of the SLD did not show any significant difference in the degree of SLD overall but demonstrated that the BES was superior to the PES (P=0.002) and the SES (P=0.006). Coupled with the conclusion from previous clinical events (29), BPDES did not significantly alter the reliability following stent implantation with the application of biodegradable polymer compared with PPDES. In the analysis of angiographic outcomes, high heterogeneity was found in these trials. This phenomenon prompted caution regarding the angiographic conclusion.

BPDES were proposed to improve the long-term safety of DES as they was designed to reduce the incidence of LST and VLST. Recently, a long-term outcome of a pooled analysis of BPDES versus PPDES in patients with diabetes from three RCTs showed that BPDES were associated with comparable overall clinical outcomes at a four-year follow-up, and rates of DpST were significantly lower with BPDES (30). In the present meta-analysis, the incidence of MACE and DpST was not found to be significantly different between BPDES and PPDES on long-term follow-up. By comparing the analysis data between long- and one-year follow-up results, the coronary stenosis patients with BPDES implantation had a tendency towards a lower incidence of clinical events, particularly DpST (P=0.05). In the RCTs with a >12 month follow-up period, two trials (13,26) had a follow-up period of ≤24 months. In the subgroup analysis on the incidence of DpST, as expected, >36 months following stent implantation, BPDES showed a beneficial effect on the reduction of DpST episodes compared with the use of PPDES. This provided evidence for the correct basic mechanism used in BPDES design: A reduction in the time the polymer is in contact with tissue reduced the incidence of DpST. In addition, this result suggested that re-endothelialization was important for reducing the risk of DpST. As mentioned previously, however, in the included RCTs, only four RCTs had a follow-up period >24 months. In these four RCTs, only two RCTs had a follow-up period >36 months; therefore the conclusion based on trials with a long-term follow up requires further research for confirmation.

The main deficiencies in the present study are as follows: Firstly, the inclusion criteria did not specifically subdivide the lesion types of the patients in detail; therefore, the study lacked specific targets. As a result, the type of coronary stenosis the BPDES were more suitable for could not be determined; thus, it was not possible to provide a specific recommendation for clinical practice. Secondly, only four RCTs had a follow-up period >12 months and only two out of four RCTs had a follow-up period >36 months. The limited data did not make the analysis very reliable; the conclusion may be regarded as a reference with further research required to confirm the results.

In conclusion, the results of the present meta-analysis suggest that BPDES were noninferior to PPDES in short-term results but superior to PPDES in long-term results. According to the clinical outcomes and angiographic outcomes at short-term follow-up, BPDES and PPDES exhibited no significant differences overall but the BES was superior to the PES. Subgroup analysis, however, demonstrated that the BES with a biodegradable polymer was superior to the PES with a permanent polymer. In this first meta-analysis comparing the data from long-term follow-up, the BPDES were found to exhibit an increased safety profile over time, particularly for incidences of LST and VLST in which the BPDES were superior to PPDES. This was consistent with the original purpose of BPDES design. Additional prolonged follow-up data, however, is required for an adequate comparison of the safety and efficacy between BPDES and PPDES to be conducted, in order to provide adequate and strong evidence for the selection of stents in clinical practice.

## Figures and Tables

**Table I:** Main clinical characteristics of the trials.

			Stent type		No. of patients (lesions)		Mean age^a^, years		Male, %	
						
First author, year (ref)	Trial acronym	Event location	BPS	PPS	BPS	PPS	BPS	PPS	BPS	PPS
Byrne, 2009 (10) Byrne, 2011 (11) Kufner, 2011 (19)	ISAR-TEST IV	Munich, Germany	Sirolimus	Sirolimus or everolimus	1299 (1689)	1304 (1683)	66.7±10.7	66.8±11.1	78.2	81.7
Byrne, 2009 (12) Mehilli, 2008 (20)	ISAR-TEST III	Munich, Germany	Sirolimus	Sirolimus	202 (239)	202 (242)	66.5±11.6	65.0±10.7	75.3	76.8
Chevalier, 2007 (13)	Nobori I	Europe, Asia, Australia	Biolimus	Paclitaxel	85 (95)	35 (42)	65.0±11.0	63.0±11.0	69.0	66.0
Chevalier, 2009 (14)	Nobori I phase 2	Europe, Asia, Australia	Biolimus	Paclitaxel	153 (174)	90 (98)	62.7±10.3	63.2±11.2	74.5	68.9
Christiansen, 2013 (15)	SORT OUT V	Western Denmark	Biolimus	Sirolimus	1229 (1532)	1239 (1555)	65.0±10.6	65.2±10.3	74.6	75.1
Gao, 2013 (16)	TARGET I	China	Sirolimus	Everolimus	227	231	58.7±9.4	59.6±9.4	69.2	68.4
Kadota, 2012 (17)	-	Japan	Biolimus	Sirolimus	198 (218)	137 (150)	67.1±10.3	67.7±9.3	71.6	72.0
Krucoff, 2008 (18)	COSTAR II	USA, Belgium, Germany, New Zealand	Paclitaxel	Paclitaxel	989 (1212)	686 (846)	63.5±10.8	63.7±10.6	73.1	71.1
Natsuaki, 2013 (21)	NEXT	Japan	Biolimus	Everolimus	1617 (2059)	1618 (2010)	69.1±9.8	69.3±9.8	77.0	77.0
Smits, 2013 (23)	COMPARE II	Europe	Biolimus	Everolimus	1795 (2638)	912 (1387)	63.0±11.1	62.7±11.0	74.4	74.3
Windecker, 2008 (24) Serruys, 2013 (22)	LEADERS	Europe	Biolimus	Sirolimus	857 (1256)	850 (1213)	64.6±10.8	64.5±10.7	75.0	74.6
Zhang, 2013 (25)	-	China	Sirolimus	Sirolimus	341	321	67.5±9.8	65.9±11.1	69.2	68.5

a Mean age ± standard deviation.

BPS, biodegradable polymer stents; PPS, permanent polymer stents; ISAR-TEST, Intracoronary Stenting and Angiographic Results-Test Efficacy of 3 Limus-Eluting Stents; Nobori, randomized comparison of the Nobori Biolimus A9-eluting coronary stent with the Taxus Liberté paclitaxel-eluting coronary stent; SORT OUT, Scandinavian Organization for Randomized Trials with Clinical Outcome; COSTAR II, Cobalt Chromium Stent with Antiproliferative for Restenosis II; COMPARE II, Abluminal Biodegradable Polymer Biolimus-Eluting Stent versus Durable Polymer Everolimus-Eluting Stent; LEADERS, Limus Eluted from A Durable vs ERodable Stent coating.

**Table II:** Main angiographic baseline characteristics of the included trials.

	LAD, n (%)		LCX, n (%)		RCA, n (%)		RD^a^, mm		MLD^a^, mm		Lesion length^a^, mm	
						
First author, year (ref)	BPS	PPS	BPS	PPS	BPS	PPS	BPS	PPS	BPS	PPS	BPS	PPS
Byrne, 2009 (10) Byrne, 2011 (11) Kufner, 2011 (19)	753 (44.7)	748 (44.3)	454 (27.0)	453 (26.8)	476 (28.3)	488 (28.9)	2.79±0.47	2.80±0.52	0.98±0.50	0.98±0.51	14.8±8.6	15.0±8.8
Byrne, 2009 (12) Mehilli, 2008 (20)	110 (46.0)	104 (43.0)	53 (22.2)	69 (28.5)	76 (31.8)	69 (28.5)	2.74±0.52	2.75±0.51	1.06±0.42	1.13±0.49	13.9±7.2	14.6±7.0
Chevalier, 2007 (13)	52 (54.7)	28 (54.8)	21 (22.1)	9 (19.0)	22 (23.2)	13 (26.2)	2.70±0.44	2.71±0.52	1.06±0.24	1.12±0.38	11.35±4.51	11.03±4.75
Chevalier, 2009 (14)	62 (35.6)	46 (46.9)	41 (23.6)	19 (19.4)	71 (40.8)	33 (33.7)	NA	NA	NA	NA	NA	NA
Christiansen, 2013 (15)	623 (40.7)	636 (40.9)	355 (23.2)	350 (22.5)	508 (33.2)	535 (34.4)	3.2±0.34	3.3±0.33	NA	NA	18.0±3.75	18.00±4.50
Gao, 2013 (16)	147 (64.8)	139 (60.2)	39 (17.2)	42 (18.2)	41 (18.1)	50 (21.6)	2.87±0.47	2.90±0.50	0.96±0.40	0.95±0.42	15.7±7.1	15.7±6.7
Kadota, 2012 (17)	83 (39.9)	62 (44.7)	56 (27.1)	33 (24.0)	68 (33.0)	43 (31.3)	2.68±0.57	2.68±0.54	NA	NA	12.64±5.52	12.82±6.81
Krucoff, 2008 (18)	528 (39.9)	529 (40.3)	369 (27.9)	402 (30.6)	426 (32.2)	383 (29.2)	2.77±0.47	2.75±0.48	0.86±0.40	0.89±0.41	15.4±6.5	15.1±6.5
Natsuaki, 2013 (21)	795 (49.0)	774 (48.0)	405 (25.0)	435 (27.0)	552 (34.0)	517 (32.0)	2.62±0.60	2.61±0.57	0.77±0.44	0.75±0.42	19.5±12.8	19.3±13.1
Smits, 2013 (23)	1078 (40.9)	550 (39.7)	602 (22.8)	356 (25.7)	882 (33.4)	448 (32.3)	2.9±0.5	2.9±0.5	NA	NA	16.8±9.8	17.7±10.6
Windecker, 2008 (24) Serruys, 2013 (22)	467 (37.2)	482 (39.7)	352 (28.0)	286 (23.6)	386 (30.7)	399 (32.9)	2.60±0.61	2.60±0.57	0.91±0.50	0.95±0.52	12.7±8.1	12.4±8.5
Zhang, 2013 (25)	216 (48.4)	217 (53.0)	90 (20.2)	65 (20.3)	140 (31.4)	129 (31.5)	NA	NA	NA	NA	29.2±16.6	24.8±14.5

a Data presented as the mean ± standard deviation.

LAD, left anterior descending artery; LCX, left circumflex artery; RCA, right coronary artery; RD, reference diameter; MLD, minimal lumen diameter; BPS, biodegradable polymer stent; PPS, permanent polymer stent.

**Table III:** Results evaluated by the GRADE system.

A, Cardiac events, target lesion revascularization and thromobosis					

Outcomes	Comparative risks^a^ (95% CI)		Relative effect (95% CI)	No. of participants (no. of studies)	Quality of evidence (GRADE)

	Assumed risk PPS per 1000	Corresponding risk BPS per 1000			
Major adverse cardiac events at one-year follow-up^a^			OR 1.05 (0.93–1.18)	15938 (11)	++++ (high)
Study population	76	80 (71–89)			
Moderate	63	66 (58–74)			
Target lesion revascularization at one-year follow-up^b^			OR 0.98 (0.84–1.15)	14263 (10)	+++− (moderate)
Study population	47	46 (39–53)			
Moderate	40	39 (34–46)			
Definite or portable stent thrombosis at one-year follow-up^b^			OR 1.03 (0.73–1.46)	15818 (10)	+++− (moderate)
Study population	8	8 (6–12)			
Moderate	3	3 (2–4)			
Major adverse cardiac events at long term follow-up^c^			OR 0.89 (0.78–1.02)	5376 (4)	++++ (high)
Study population	143	128 (112–146)			
Moderate	127	113 (99–130)			
Target lesion revascularization at long term follow-up^d^			OR 0.92 (0.78–1.07)	5376 (4)	++++ (high)
Study population	110	101	(86–118)		
Moderate	107	98	(83–114)		
Definite stent or portable thrombosis at long term follow-up^c^			OR 0.72 (0.51–1.04)	5396 (4)	++++ (high)
Study population	27	20 (14–28)			
Moderate	17	12 (9–18)			

B, Late lumen loss and stenosis					

Outcomes	Absolute difference in outcome		No. of participants		Quality of evidence (GRADE)

Late lumen loss in stent (mm)^e^	Mean late lumen loss in stent in the intervention groups was 0.04 (0.08–0.00) lower		5173 (8)		++−− (low)
Stenosis of lumen diameter in stent (%)^e^	Mean stenosis of lumen diameter in stent in the intervention groups was 1.43 lower (3.02 lower to 0.17 higher)		5183 (8)		++−− (low)

The corresponding risk (and its 95% CI) is based on the assumed risk in the comparison group and the relative effect of the intervention (and its 95% CI).

a follow-up of 12 months;

b–e mean follow-ups of ^b^12, ^c^36, ^d^33 and ^e^8 months;

CI, confidence interval; OR, odds ratio; GRADE, GRADEPro software (Cochrane Informatics and Knowledge Managment, London, UK). PPS, permanent polymer stent; BPS, biodegradable polymer stent.
